# The evaluation of off-loading using a new removable oRTHOsis in DIABetic foot (ORTHODIAB) randomized controlled trial: study design and rational

**DOI:** 10.1186/s13047-016-0163-4

**Published:** 2016-08-22

**Authors:** Kamel Mohammedi, Louis Potier, Maud François, Dured Dardari, Marilyne Feron, Estelle Nobecourt-Dupuy, Manuel Dolz, Roxane Ducloux, Abdelkader Chibani, Dominique-François Eveno, Teresa Crea Avila, Ariane Sultan, Laurence Baillet-Blanco, Vincent Rigalleau, Gilberto Velho, Florence Tubach, Ronan Roussel, Jean-Claude Dupré, Dominique Malgrange, Michel Marre

**Affiliations:** 1 Department of Endocrinology, Diabetes, and Nutrition, DHU FIRE, Bichat-Claude Bernard Hospital, Assistance Publique Hôpitaux de Paris, Paris, France; 2 INSERM, UMR_S 1138, Centre de Recherche des Cordeliers, Paris, France; 3 Centre Hospitalier Universitaire de Reims, Service d’Endocrinologie, Diabète, Nutrition, Reims, France; 4 Department of Diabetology, Endocrinology and Nutrition, Centre hospitalier Sud Francilien, Corbeil-Essonnes, France; 5 Department of Diabetology, Endocrinology and Nutrition, Centre Hospitalier Universitaire de Nantes, Nantes, France; 6 Endocrinology Department, Hôpital Bégin, Saint-Mandé, France; 7 APHP, Hôpital Corentin-Celton, Centre de Cicatrisation du Pied du Diabétique, Issy-les-Moulineaux, France; 8 Department of Diabetology, Endocrinology and Nutrition, Centre Hospitalier de Gonesse, Gonesse, France; 9 Department of functional rehabilitation, Centre Hospitalier La Tourmaline, Saint Herblain, France; 10 Department of Diabetology, Centre Hospitalier Régional de Metz - Thionville, Endocrinology and Nutrition, Thionville, France; 11 Endocrinology-Diabetology-Nutrition Department, CHRU Montpellier, Montpellier, France; 12 INSERM U1046, University of Montpellier 1, Montpellier, France; 13 Nutrition and Diabetology Unit, CHU de Bordeaux, Haut Lévèque Hospital, Pessac, France; 14 University of Bordeaux, Bordeaux, France; 15 Département d’Epidémiologie et Recherche Clinique, APHP, CIC-EC 1425, Centre de Pharmacoépidémiologie (Cephepi), Paris, France; 16 Université Paris Diderot, Sorbonne Paris Cité, UFR de Médecine, Paris, France

**Keywords:** Diabetic foot, Wound, Healing, Off-loading, Device

## Abstract

**Background:**

Off-loading is essential for diabetic foot management, but remains understudied. The evaluation of Off-loading using a new removable oRTHOsis in DIABetic foot (ORTHODIAB) trial aims to evaluate the efficacy of a new removable device “*Orthèse Diabète*” in the healing of diabetic foot.

**Methods/design:**

ORTHODIAB is a French multi-centre randomized, open label trial, with a blinded end points evaluation by an adjudication committee according to the Prospective Randomized Open Blinded End-point. Main endpoints are adjudicated based on the analysis of diabetic foot photographs. *Orthèse Diabète* is a new removable off-loading orthosis (PROTEOR, France) allowing innovative functions including real-time evaluation of off-loading and estimation of patients’ adherence. Diabetic patients with neuropathic plantar ulcer or amputation wounds (toes or transmetatarsal) are assigned to one of 2 parallel-groups: *Orthèse Diabète* or control group (any removable device) according to a central computer-based randomization. Study visits are scheduled for 6 months (days D7 and D14, and months M1, M2, M3, and M6). The primary endpoint is the proportion of patients whose principal ulcer is healed at M3. Secondary endpoints are: the proportion of patients whose principal ulcer is healed at M1, M2 and M6; the proportion of patients whose initial ulcers are all healed at M1, M2, M3, and M6; principal ulcer area reduction; time-related ulcer-free survival; development of new ulcers; new lower-extremity amputation; infectious complications; off-loading adherence; and patient satisfaction. The study protocol was approved by the French National Agency for Medicines and Health Products Safety, and by the ethics committee of Saint-Louis Hospital (Paris). Comprehensive study information including a Patient Information Sheet has been provided to each patient who must give written informed consent before enrolment. Monitoring, data management, and statistical analyses are providing by UMANIS Life Science (Paris), independently to the sponsor. Since 27/10/2013, 13 centres have agreed to participate in this study, 117 participants were included, and 70 have achieved the study schedules. The study completion is expected for the end of 2016, and the main results will be published in 2017.

**Conclusion:**

ORTHODIAB trial evaluates an innovating removable off-loading device, seeking to improve diabetic foot healing (ClinicalTrials.gov identifier: NCT01956162).

**Electronic supplementary material:**

The online version of this article (doi:10.1186/s13047-016-0163-4) contains supplementary material, which is available to authorized users.

## Background

Diabetic foot is a major public health problem worldwide [1]. It is associated with poor survival and functional outcomes with a high rate of recurrence [2–6]. Diabetic foot is a leading cause of lower-extremity amputation [2] with fifty percent of non-traumatic amputations practiced in patients with diabetes [7, 8]. Diabetic foot is characterized by a slow and difficult healing with high risk of amputation and infectious disease [5]. Increased plantar pressure is the most important factor for the development of plantar ulcers in diabetic patients [9, 10].

Off-loading is a key therapeutic technique essential to managing patients with neuropathic diabetic foot. It improves healing by reducing the disproportionate pressure points on the wound [11]. Previous randomized controlled trials (RCT) have confirmed the importance of off-loading to heal diabetic foot ulcers [12, 13]. There are two major off-loading systems using a removable or a non-removable device. The total contact cast (TCC) is the most efficient device, and considered as the gold standard for diabetic foot off-loading [13, 14]. Removable devices are easier to prescribe, with improved acceptability to patients, but they do not always achieve a satisfactory healing [15–17]. The evaluation of Off-loading using a new removable oRTHOsis in DIABetic foot (ORTHODIAB) trial aims to evaluate the efficacy of a new customized removable plantar off-loading device “*Orthèse Diabète*” in the healing of neuropathic diabetic foot. We report here the design and rational of ORTHODIAB trial according to the 2013 guidelines of the Standard Protocol Items: Recommendations for Interventional Trials (SPIRIT) [18].

## Methods

### Study design

ORTHODIAB is a French collaborative multi-centre randomized, open label trial, with a blinded end points evaluation by an adjudication committee according to the Prospective Randomized Open Blinded End-point (PROBE) method [19].

### Participants

The main eligible criteria are (i) age over 18 years; (ii) diagnosis of type 1 or type 2 diabetes based on the American Diabetes Association experts consensus [20]; (iii) sensory peripheral neuropathy (defined as abnormal 10 g-monofilament test, i.e., not perceived at least 2 times in 1 of the 3 areas explored: pulp of the big toe, 1st and 5th metatarsal heads) [21]; and (iv) one or more plantar ulcerations or lower-extremity amputation wounds (toes or transmetatarsal). To exclude patients for whom off-loading is not crucial, severe peripheral arterial disease (defined as ankle-brachial index < 0.7, or transcutaneous oxygen pressure < 30 mm Hg, or great toe pressure < 30 mm Hg) [22], and severe skin or bone infection (requiring parenteral antibiotic therapy or surgery) are considered as non-inclusion criteria. The other exclusion criteria are a large ulcer in the homolateral leg (> 20 cm^2^ of area), contralateral above heel amputation, overweight (>130 Kg), pregnancy or the likelihood of pregnancy, persons under guardianship, and persons with loss of functional and/or neuropsychological autonomy.

## Devices and study allocation

### Experimental device

**Fig. 1:**
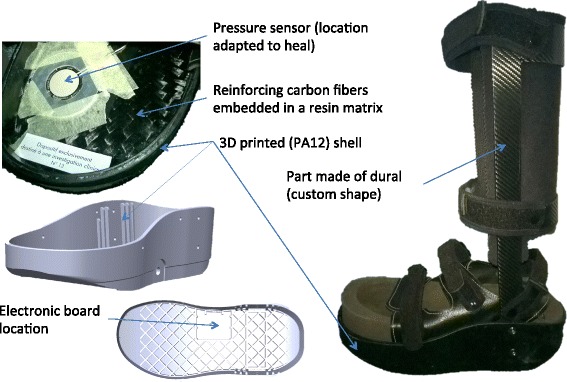
Schematic illustration of “Orthèse Diabète” and its different components

*Orthèse Diabète* allows off-loading through elimination of the weight bearing on the plantar wound and limitation of the shearing forces. This new device offers two additional innovative connected features: (i) evaluation of the pressure applied on the wound using a non-invasive and repeating sound system; and (ii) a thermal sensor measuring the leg temperature that allows an estimation of the real time adherence to the off-loading. A schematic illustration of *Orthèse Diabète* and its different components is shown in Fig. 1.

### Components and technical characteristics of the experimental device

“Orthèse Diabète” weighs 1.294 kg. Its innermost part (foot interface) is made up of a foot part (insole) and a circumferential ridge (side and anteroposterior protections of the foot). This foot interface is designed from a scan of the foot, which is then integrated into a specific CAD-CAM. Each foot interface is realized with its own geometrical and mechanical properties (specific hardness of the EVA). The principle of the off-loading relies on the excavation of the area facing the wound, and a redistribution of the load in the healthy areas. The theoretical optimization of the pressure field under the foot is confirmed by a pressure measurement function permanently integrated in the orthosis. If the wound is not correctly offloaded during walking an audible alarm sounds. During fitting and outpatient clinics, the control of the off-loading of the wound is achieved thanks to this function. The internal battery provides power for at least 90 days of autonomous measurement when it is turned-on permanently. The orthotist can modify the foot interface until the absence of alarm. If this feature is permanently enabled, it also allows the detection of a decrease of the off-loading efficiency. If it happens, the patient can modify his gait pattern to stop the ringing and should consult his healthcare team as soon as possible. In the ORTHODIAB protocol, it was decided to turn this function off between study visits.

A hard plastic shell protects the foot interface, integrates the electronic components, and constitutes the ankle joint due to its junction with the upright. To allow healing, the brace system locks all joints of the foot and ankle in a position of 3° of dorsiflexion of the foot. This posture allows a smooth transition between the mid stance and the terminal stance. This immobilization of the foot joints is an important feature because it limits the stretching of soft tissue and relieves pressure, shear, and friction on the foot skin. However, this immobilization decreases very significantly the quality of the stance phase during gait and generates overpressure on the anterior part of the leg. To counterbalance the loss of mobility of the foot and maintain a comfortable walking, the device has an outsole with a moderate roll over shape. The form of the rollover is a compromise between quality of walking from one side and instability, height of the device on the other side. The outsole material is a non-marking rubber with variable traction pattern. The balance of the pelvis is kept by a compensation insole in the shoe of the contralateral limb.

The first criterion of success for the wound healing is that the off-loading is achieved. Thus, to be effective, the orthosis must be worn at all times with the exception of sleep and rest periods. A monitoring function (thermal sensor) measuring the time of worn is integrated into the brace to help the medical team and the patient to quantify the use. In addition to the use of the orthosis, technical aids (one or two crutches) are recommended to improve offloading and the stability of walking. However, it is not recommended to the patient to perform prolonged or heavy physical activities, especially in rough, wet or muddy ground.

### Control group

Any standard or customized removable off-loading device allowable and available in France. The choice of the conventional device is based on the local practices of each centre.

### Study endpoints

The primary endpoint is the proportion of patients whose principal ulcer is fully healed at the 3-month time-point. The principal ulcer is defined as a unique ulcer, or the largest one if the patient has multiple (≥2) ulcerations at baseline. Secondary endpoints are defined as the proportion of patients whose principal plantar ulcer is fully healed at 1, 2 and 6 months respectively; the proportion of patients with all initial plantar ulcers fully healed at 1, 2, 3 and 6 months; the percentage area reduction of the principal plantar ulcer at 1, 2, 3 and 6 months; time-related principal ulcer-free survival; the development of new ulcers; new cases of lower-extremity amputation; incidence of infectious complications; adherence to off-loading; and patient satisfaction with the prescribed device. The primary and the first four secondary endpoints are adjudicated by an independent End Point Adjudication Committee blinded to the study allocation, centres, and investigators. The remaining endpoints are collected systematically from all participants during scheduled study visits using case report forms, and from reports of adverse events (AEs). AEs and serious AEs (SAE) are monitored independently by the department of pharmacovigilance of the university hospital of Dijon (Centre Régional de Pharmacovigilance de Bourgogne, Pôle des Pathologies Lourdes et des Vigilances – CHU Le Bocage, Dijon, France).

### Measurment procedure

At every visit, investigators take digital images of plantar ulcers after debridement of the wound and the periwound hyperkeratosis [23]. Two digital images are required for each ulcer; one focused on the ulcer and the other on the whole foot. The same brand and model of digital camera (*Panasonic®* DMC- TZ 25), as well as the same photographic technique and settings (Flash: on, size of picture: 2048 × 1536 pixels (3 M), electronic image stabilizer: on, and autofocus uses a small area located in the centre of image to determine focus) are used by each centre. After their teletransmission to the contract research organization (UMANIS Life Science, Paris, France), the photographs are blinded and transferred to the Adjudication Committee. Each member of the Adjudication Committee measures the unhealed areas of the plantar ulcers using a specialized software *(Tracer.exe,* university of Glamorgan, UK) [24], and determines the healing status (see “Procedures of adjudication”). Full healing is defined as complete epithelialization of the wound. Observance of off-loading is evaluated in the two study groups using a semi-quantitative questionnaire administered to patients at each visit. This questionnaire evaluates the frequency (always, often, sometimes, or rarely) of off-loading device use, whether inside or outside the house and if during the night. Furthermore, the thermal sensor is examined in the *Orthèse Diabète* group. Patient satisfaction is evaluated 3 months after device provision using the Quebec User Evaluation of Satisfaction with Assistive Technology (QUEST) survey.

### Randomization and study schedules

**Fig. 2:**
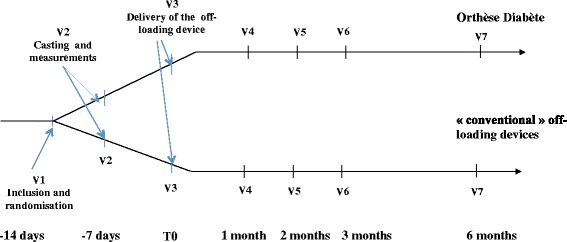
Schedule of the study visits

In addition to provision of appropriate wound care, study participants are randomly assigned to one of 2 parallel-groups: *Orthèse Diabète* or conventional device according to a central computer-based 1:1 randomization without stratification. The allocation sequence is generated by the contract research organization. Participants are enrolled and assigned to their groups by the responsible investigator. Device measurement and casting are scheduled 7 days after randomization, and the off-loading system is delivered 7 days later. Follow-up visits are scheduled 1, 2, 3 and 6 months after the delivery of the device (Fig. 2). Comprehensive schedule of study procedures are given in Table 1. The recruitment period, follow-up of participants, and full study last 30, 6.5, and 36.5 months, respectively.

**Table 1:** Schedule of study procedures

	Inclusion and randomization	Casting and measurements	Delivery of the offloading device	Follow-up			
Visits	V1	V2	V3	V4	V5	V6	V7
Weeks*	0	1	2	6	10	14	26
Verification of the inclusion or non-inclusion criteria	x						
Patient Education and signature of the consent	x						
Collection of demographic and clinical data^a^	x						
Collection of the diabetes history^b^	x						
Collection of the past medical history^c^	x						
Collection of adverse events		x	x	x	x	x	x
Collection of the current treatments	x		x	x	x	x	x
Clinical examination^d^	x						
Biological measurements^e^	x						
Podiatric examination	x						
Evaluation of the principal ulcer	x		x	x	x	x	x
Local wound care	x	x	x	x	x	x	x
Evaluation of concomitant ulcer(s)	x		x	x	x	x	x
Randomization	x						
Taking photographs of the ulcer(s)	x		x	x	x	x	x
Sending the photographs to UMANIS	x		x	x	x	x	x
Collection of podological complications			x	x	x	x	x
Appearance of new ulcer(s)			x	x	x	x	x
Delivery the off-loading device			x				
Verification of the off-loading quality				x	x	x	x
Wearing frequency of the device				x	x	x	x
Obtaining an appointment	x	x	x	x	x	x	

*Weeks ± 3 days; ^a^age, sex, profession and history of tobacco smoking; ^b^Type, duration and complications of diabetes; ^c^History of podiatric, medical and surgical disease; ^d^Weight, height, heart rate, systolic and diastolic blood pressure, and podological examination; ^e^HbA1c, plasmatic creatinine, estimated Glomerular Filtration Rate computed by the Chronic Kidney Disease Epidemiology Collaboration equation, and urinary albumin-creatinine ratio

### Statistical considerations

The present study proposes to evaluate the efficacy of *Orthèse Diabète* compared to conventional off-loading devices. Based on results of a comparable published RCT, we assumed that the healing-rate will be 80 and 52 % at 3 months for *Orthèse Diabète* and a conventional removable device, respectively [15]. Accordingly, our study requires at least 110 patients (55 for each arm) to achieve a statistical power of 80 % for an alpha level of 5 % and a rate of discontinuation of 5 %. The analyses are performed according to the intention-to-treat principle, including all randomized patients, whether or not they have used the prescribed device. Sensitivity analyses will be performed in participants who have used the study device for at least 2 weeks. No interim analyses are planned. Statistical analyses will be performed using SAS software, version 9.3 (SAS Institute, www.sas.com).

### Procedures of adjudication

Accordance between pairs of experts on full healing is assessed using Cohen’s Kappa coefficients. Because perfect accordance (Kappa = 1) is required for this primary endpoint, each discordant case is reviewed during an Adjudication Committee meeting to establish a consensual decision. In case of persistent disagreement, the most represented value is retained. Intraclass correlation coefficients (ICC agreement and ICC consistency) are calculated to evaluate global agreement for the ulcer area. Absolute and relative differences calculations complete inter-rater reliability appraisal, and Bland-Altman plots lay out measures dispersion. The average of the measurements will be retained for each ulcer. In case of important mean relative difference between the three measures (15 to 20 %), the mean of the two nearest values is retained. Where there is over 20 % relative difference for each pair of experts, a consensual decision is made through the Adjudication Committee, or the median value is retained in case of persistent discrepancy.

### Study organization and progress

Thirteen centres in France have agreed to participate in the ORTHODIAB trial. The list of centres and investigators is provided in the online Additional file 1. Participant recruitment started 27 October 2013 and ended 27 May 2016. Over the recruitment period, 128 patients were assessed for eligibility with 117 randomly assigned. Of the 117 randomised, 70 have already achieved the whole study schedule. The follow-up visits will continue until the end of December 2016, and the main results will be published in 2017. Monitoring, data management, and statistical analyses are providing by UMANIS Life Science (Paris, France), independently to the sponsor and competing interests.

## Discussion

This report describes the rationale and design of the ORTHODIAB trial aiming to evaluate the efficacy of *Orthèse Diabète,* a new removable off-loading device for the healing of neuropathic plantar ulcers in patients with diabetes.

Despite its prevalence, severity, and high social and economic burden, research into diabetic foot is limited and poorly funded. [25]. Care costs of patients with diabetic foot are high, representing about 33 % of total costs related to all care of patients with diabetes [26]. Diabetic foot management and limb salvage require considerable efforts with specialized therapies such as revascularization procedures, skin and bone infection management, and advanced wound-healing modalities including off-loading. As a non-removable device, TCC allows better healing, especially because adherence to off-loading is mandatory. However, technical difficulties limit TCC use. A large survey performed in 895 podiatry clinics managing diabetic foot ulcers in the USA revealed that only 1.7 % used non-removable off-loading devices [27]. Fife et al. reported also that non-removable devices were used by only 6 % of patients treated for foot ulcers [28]. Furthermore, 29 % of amputee patients stopped wearing a TCC before the end of the period recommended for off-loading because of side effects [29]. On the other hand, a removable cast walker made irremovable had similar efficacy to TCC, and was faster to implement, easier to use, and less expensive in diabetic patients with plantar ulcers [12]. It is therefore noteworthy that we are evaluating in the present study a new removable device equipped with a thermal sensor helping to improve the observance of off-loading. A Previous study had confirmed the interest and reliability of a thermal data-logger within a spinal orthosis in patients with idiopathic scoliosis [30]. *Orthèse Diabète* is also equipped with a non-invasive and repeating sound system allowing real-time evaluation of off-loading during device casting.

Blinded studies evaluating off-loading in patients with diabetic foot are impossible to fulfil, because of different and ostensible aspects of devices. To reduce methodological bias in the present trial, we are using a PROBE method allowing comparable results with a double blind design [19]. The End Point Adjudication Committee will measure the area on each plantar ulcer, using photographs of the ulcer with Digital Photo Planimetry software [24]. The photographic method allows reliable measurements of wound surface area through scaling and manual cropping of the digital photography [31].

The main strengths of our study is the randomization of more than 110 patients with neuropathic diabetic foot in 13 diabetes centres across France, the collection of their clinical and digital image data, and the blinded adjudication of the key outcomes. We also test a new removable off-loading device with innovative connected functions allowing a real-time evaluation of the off-loading quality, and adherence of patients. The principal limitation of the present trial is the use of different removable devices in the control group. The absence of standardisation of wound care across study centres could be another limitation. However, the possible bias related to the absence of such standardisation is reduced by the recommendation of systematic wound and peri-wound debridement, whose efficiency is clearly supported by several studies [23].

In conclusion, ORTHODIAB is an ongoing RCT conducted in 13 diabetes centres in France, testing a new technically innovative removable off-loading device in patients with diabetes and neuropathic plantar ulcers. This study might provide evidence for the interest of a real-time evaluation of the off-loading quality and adherence in the management of diabetic foot.

## Additional file

Additional file 1: Members of the ORTHODIAB Collaborative Group. (DOCX 56 kb)

## Abbreviations

AEs: Adverse events
*ANSM*: French national agency for medicines and health products safety agency
ICC: Intraclass correlation coefficients
ORTHODIAB: Evaluation of Off-loading using a new removable oRTHOsis in DIABetic foot
PROBE: Prospective randomized open blinded end-point
QUEST: Quebec user evaluation of satisfaction with assistive technology survey
RCT: Randomized controlled trials
SAE: Serious AEs
SPIRIT: Standard protocol items: recommendations for interventional trials
TCC: Total contact cast
