# Supplementary material for: The evaluation of off-loading using a new removable oRTHOsis in DIABetic foot (ORTHODIAB) randomized controlled trial: study design and rational
Source: J Foot Ankle Res. 2016 Aug 22;9(1):34. doi: 10.1186/s13047-016-0163-4 (PMC4994157; doi:10.1186/s13047-016-0163-4)
Supplement: Additional file 1: — Members of the ORTHODIAB Collaborative Group. (DOCX 56 kb) [file 13047_2016_163_MOESM1_ESM.docx]

**Supplementary Appendix Material**

**1. Members of the ORTHODIAB Collaborative Group**

**List of investigators:** K. Mohammedi and L. Potier (Paris); M. François (Reims); D. Dardari (Corbeil-Essonnes); E. Nobecourt-Dupuy (Nantes); L. Bordier and M. Dolz (Saint-Mandé); R. Ducloux (Issy-les-Moulineaux); A. Chibani (Gonesse); D.F. Eveno and M. Dacosta (Saint-Herblain); T. Crea Avila and C. Mouget (Thionville); A. Sultan (Montpellier); T. Louissaint (Créteil); L. Baillet-Blanco and V. Rigalleau (Bordeaux); N. Jourdan (Nîmes).

**End Point Adjudication Committee:** Q. Pellenc (Paris), J.C. Dupré (Paris), D. Malgrange (Reims).

**Scientific committee:** K. Mohammedi (Paris), E. Dechamps (Paris), R. Roussel (Paris), J.C. Dupré (Paris), D. Malgrange (Reims), M. Marre (Paris).
